# Differential between Protein and mRNA Expression of CCR7 and SSTR5 Receptors in Crohn's Disease Patients

**DOI:** 10.1155/2009/285812

**Published:** 2010-02-01

**Authors:** Nathalie Taquet, Serge Dumont, Jean-Luc Vonesch, Didier Hentsch, Jean-Marie Reimund, Christian D. Muller

**Affiliations:** ^1^Laboratoire d'Innovation Thérapeutique, UMR 7200, Faculté de Pharmacie, Université de Strasbourg, 74 Route du Rhin, B.P. 24, 67401 Illkirch Cedex, France; ^2^Centre d'Imagerie, Institut de Génétique et de Biologie Moléculaire et Cellulaire, INSERM U596, CNRS UMR7104, Université Louis Pasteur, 1 Rue Laurent Fries, 67404 Illkirch Cedex, France; ^3^IFR 146 ICORE, EA 3919 (Biologie Moléculaire et Cellulaire de la Signalisation), Université de Caen—Basse Normandie, Hôpital Côte de Nacre, UFR de Médecine, 14032 Caen Cedex, France; ^4^Service d'Hépato-Gastro-Entérologie et Nutrition, Pôle Reins-Digestif-Nutrition, Centre Hospitalier Universitaire, Avenue de la Côte de Nacre, B.P. 95182, 14033 Caen Cedex 09, France

## Abstract

Crohn's disease (CD) is a multifactorial chronic inflammatory bowel disease of unknown cause. The aim of the present study was to explore if mRNA over-expression of SSTR5 and CCR7 found in CD patients could be correlated to respective protein expression. When compared to healthy donors, SSTR5 was over-expressed 417 ± 71 times in CD peripheral blood mononuclear cells (PBMCs). Flow cytometry experiments showed no correlation between mRNA and protein expression for SSTR5 in PBMCs. In an attempt to find a reason of such a high mRNA expression, SSTR5 present on CD PBMCs were tested and found as biologically active as on healthy cells. In biopsies of CD intestinal tissue, SSTR5 was not over-expressed but CCR7, unchanged in PBMCs, was over-expressed by 10 ± 3 times in the lamina propria. Confocal microscopy showed a good correlation of CCR7 mRNA and protein expression in CD intestinal biopsies. Our data emphasize flow and image cytometry as impossible to circumvent in complement to molecular biology so to avoid false interpretation on receptor expressions. Once confirmed by further large-scale studies, our preliminary results suggest a role for SSTR5 and CCR7 in CD pathogenesis.

## 1. Introduction

Attempts to correlate protein abundance with mRNA expression levels have had variable success. Three main reasons have been suggested for the poor correlations between mRNA and protein levels generally reported in the literature [1]. First, there are many posttranscriptional mechanisms involved in turning mRNA into proteins: a number of complex steps between transcription and translation occur. Second, proteins may differ substantially in their in vivo half-lives: the cell can control the rates of degradation or synthesis for a given protein, and there is significant heterogeneity even within proteins that have similar functions. Third, there is a significant amount of error and background noise in both protein and mRNA experiments that limit our ability to get a clear picture. The soundest way to follow protein expression is the use of an antibody able to tag specifically the desired epitop. This is applied generally in western blot but can be more rapidly revealed by cytometry (image or flow). To test the pertinence of confocal microscopy and flow cytometry, we used these two techniques to determine protein abundance of somatostatin receptor 5 (SSTR5) and chemokine receptor CCR7, two receptors that we have found to be significantly increased in Crohn's disease (CD) patients' peripheral blood mononuclear cells (PBMCs) and inflamed intestinal mucosa, respectively (ongoing work, full data not published). Crohn's disease is a chronic inflammatory bowel disease, which can affect the whole gut, but is more often located to the distal part of the small intestine (ileum) and/or to the colon. Current pathogenic hypotheses suggest that CD results from an aberrant immune response towards (a) bacteria(s) from the gut flora, in genetically susceptible hosts [2, 3]. Nevertheless, and despite numerous works examining specific potential pathogenic pathways or analyzing pan-genome modifications in CD which have been performed, results are often disappointing and sometimes contradictory or not reproducible from one study to another. This probably depends both on patient populations heterogeneity and variability, and on the methodological and technical approach used. Therefore, identifying the most reliable biochemical and/or biological techniques to study the fundamental features of CD appears to be a preliminary work of outstanding importance.

The aim of the present study was to explore if the differential mRNA expression of SSTR5, we observed in PBMCs from CD patients, and that of CCR7 in CD patients biopsies, could be correlated to protein expression monitored by flow or image cytometry.

## 2. Materials and Methods

### 2.1. Crohn's Disease Patients

Ten patients aged 28 (median, range: 20–42; 6 women, 4 men) presenting colonic (*n* = 4) or ileocolonic (*n* = 6) disease participated to the study. At the time tissue samples (blood or intestinal mucosa) were collected, their disease was inactive (assessed by the Harvey Bradshaw index; <4 for all patients) for more than 3 months (median: 8, range: 3–27). No patient exhibited clinical signs of infection and C-reactive protein concentration measured routinely was normal (<5 mg/L) in all patients. Finally, stool culture, performed systematically, was negative for the whole group. Two patients were treated on the long term by infliximab, 3 patients by azathioprine, one by methotrexate, all on stable doses for more than 3 months. Four patients had no medication. Paired healthy age and sex-matched donors were chosen for comparison. This work was approved by the ethic committee (Comité de Protection des Personnes Nord-Ouest II, Amiens, France), and carried out according to national guidelines.

### 2.2. Isolation of PBMCs and Tissue Collection

Peripheral blood mononuclear cells were obtained through venopuncture, in the morning, from overnight fasting patients or healthy unpaid control volunteers. Cells were separated using Ficoll-Histopaque density 1.077 gradient centrifugation as previously described [4]. They were activated or not by lipopolysaccharide from *Salmonella abortus equi *(LPS : 1 *μ*g/mL sterile water; Sigma Chemical 100 mg, Salmonella serotype 0128:B12) for 2 hours 30 minutes at 37°C. Then, PBMCs were centrifuged, the pellet frozen in liquid nitrogen and kept at −80°C. Cell culture supernatants were frozen for cytokine determination.

Healthy control mucosal biopsies were obtained in patients undergoing colonoscopy for colorectal cancer screening (for family history of colorectal cancer; with no clinical symptoms or biological abnormalities) showing normal colonoscopy. In CD patients, biopsies were obtained during colonoscopy performed to assess disease extent, severity, and/or progression.

A sample of each tissue specimen was used for histopathological studies, and samples from the same colonic area were frozen on D solution [5] and stored at −80°C. In CD patients, biopsies have been performed in inflamed mucosa.

### 2.3. RNA Extraction

Tissues were defrosted 15 minutes on ice [5]. The cells were broken with a sterile piston in phenol saturated 2 M sodium acetate (pH 4.0) and chloroform/isoamylic alcohol (49 : 1). After vortexing, the lysates were incubated 15 minutes on ice then centrifuged for 20 minutes at 12000 g (4°C). Aqueous phase containing the RNA was saved and the RNA precipitated by isopropanol for one hour at −20°C. The RNA extract was centrifuged during 20 minutes 12000 g (4°C), and washed with 500 *μ*L of 70% ethanol, then centrifuged 5 minutes at 12000 g (4°C) and washed again with 75% ethanol. The RNA extract was dried in a Speed Vac and resuspended in sterile water.

The Tri Reagent (Molecular Research Center, Inc., Cincinnati, Ohio, USA) was used to isolate RNA from PBMCs (5 · 10^6^ cells) according to manufacturer's instructions. Total mRNA was checked for integrity and concentration by means of the RNA 6000 LabChip kit on an Agilent 2100 bioanalyzer: ratios of 28S RNA/18S RNA had to be above 1.6 to validate a sample. Purity was obtained by reading the absorbance at 260 and 280 nm ratio (A260/A280) between 1.8 and 2.0 to validate a sample. The RNA Integrity Number (RIN) for assigning integrity values to RNA measurements, was evaluated for each sample according to Schroeder et al. [6].

### 2.4. Real-Time RT-PCR and PCR

Five *μ*g of total RNA is reverse transcribed to single-stranded cDNA using the High-Capacity cDNA Archive Kit. The RNA is preincubated at 25°C for 10 minutes, followed by 2 hours at 37°C, according to the manufacturer's recommendations (Applied Biosystems, Foster City, California, USA). Subsequently, cDNA was kept at −20°C until used. In a 96 wells plate (MicroAmp Optical 96-well Reaction, Applied Biosystems), an aliquot of cDNA (100 ng/*μ*L) was mixed carefully in TaqMan Universal PCR Master Mix, DEPC water (Euromedex, Souffelweyersheim, France) and TaqMan Predeveloped assay reagents (PDAR Applied Biosystems, Foster City, California, USA) then briefly spun (15 seconds, 2000 g). The 96 wells plates were immediately sealed with an optical adhesive cover, and centrifuged twice in a Sigma centrifuge for 1 minute at 2000 g. Real-time RT-PCR has been performed using an ABI Prism 7000 (Applied Biosystems, Foster City, California, USA). Thermal cycling was carried out for 2 minutes at 50°C (to activate Uralic-DNA glycosylase), 10 minutes at 95°C (to inactivate Uralic-DNA glycosylase and activate polymerase DNA AmpliTaq Gold), and 40 cycles of 15 seconds at 95°C, then 1 minute at 60°C. Each reaction contained cDNA derived from around 1.5 ng of total RNA. The primer sequences and probes from the TaqMan Human GPCR Panel (P/N 4367785 from Applied Biosystems) are catalog no. Hs00265647_s1 for SSTR5 and Hs00171054_m1 for CCR7, respectively.

### 2.5. DNA Sequencing

After mRNA extraction, 100 pg of mRNA was added to 10 mM dNTP Mix, 2 pmoles/mL of the primers RT1 and RT2 (Operon, Köln, Germany), and sterile water. The mixture was incubated to 65°C for 5 minutes, and kept on ice. The content of the tube was collected by brief centrifugation (1000 g). The 5x First Buffer, DTT 0.1M, Recombinase Rnase Inhibitor and the SuperScript III RT (kit In Vitrogen, Cergy Pontoise, France) were then added. The tube was incubated for 5 minutes at 25°C, 60 minutes at 50°C, and 15 minutes at 70°C (PTC-100 Programmable Thermal Controller, MJ Research INC, Peltier-Effect Cycling, USA).

For one PCR tube reaction, 10x PCR Buffer (200 mM Tris-HCl pH 8.4, 500 mM KCl), MgCl_2_ 50 mM, dNTP Mix 10 mM, amplification primers PCR1 and PCR2 10 mM (Operon, Cologne, Germany), Taq DNA polymerase 5 U/mL (Phusion High Fidelity DNA Polymerase, Finnzymes, Espoo, Finland), cDNA from first-strand reaction and distilled water were mixed. The tube reaction has been put in incubation at 98°C for 30 seconds to be denatured, and during 30 cycles of 10 seconds at 98°C, for 20 seconds at 75°C, and for 1 minute at 72°C, and finish the program at 72°C during 10 minutes. DNA and SmartLadder (Eurogentec, Seraing, Belgium) were deposed in an agarose gel 1% in TAE 0.5x.

Total cDNA was checked for integrity and concentration using the DNA 7500 LabChip kit on the Agilent 2100 bioanalyzer. It was also checked for purity by reading absorbance at 260 and 280 nm: ratios A260/A280 always between 1.8 and 2.0.

### 2.6. Immunofluorescence Staining on the Tissue Paraffin Sections

Tissue paraffin sections were placed at 57°C during 15 minutes and deparaffinised according a protocol previously described by Kumada et al. [7]. Human tissue sections were stained overnight at 4°C with the primary rabbit monoclonal antibody to CCR7 1 : 250 (Abcam, Paris, France) or rabbit polyclonal to SSTR5 1 : 5000 (Ozyme, St Quentin en Yvelines, France) in incubation Buffer (0.5% BSA-TBS pH 9.0). After three passages (10 minutes) in washing buffer (0.1% BSA-TBS pH 9.0), sections were incubated for two hours in presence of the secondary goat anti-rabbit Alexa Fluor 647 antibody (In Vitrogen, Cergy Pontoise, France) in incubation buffer. Finally, they were washed for 10 minutes in Washing Buffer, then three times in TBS pH 9.0, and six times under running water. 

### 2.7. Acquisition and Images Analyzes

Image acquisition was performed using a confocal microscope Leica SP5 (LEICA, Mannheim, Germany). The software LAS AF (Leica) has been used for the acquisition and the numerical recording of the images, and its analysis has been carried out with Image J (a freeware by Wayne Rasband, National Institutes of Health, USA).

### 2.8. Flow Cytometry

Fixation and permeabilisation were performed thanks to the cytofix/cytoperm kit (BD Biosciences, Le Pont de Claix, France) and incubated one hour with a primary rabbit polyclonal antibody to SSTR5 1 : 5000 (Ozyme, St Quentin en Yvelines, France) in incubation buffer or with isotypic antibodies for all controls. After three washes in Perm/Wash, cells were incubated one hour with the Alexa 488-conjugated anti-rabbit immunoglobulin secondary antibody (Molecular Probes, Leiden, Netherlands). For all samples granulation, size, and fluorescence intensity were recorded on a FACStar + cell sorter (BD Biosciences, San Jose, CA, USA) at 800 cells/s, or on an Easycyte Plus capillary cytometer (Guava Technologies, Hayward, CA, USA) at a rate of 0.59 *μ*L/s. Data are expressed as mean fluorescence/cell; no cut off signal was applied.

### 2.9. TNF-*α* Secretion Assay

Human PBMCs from CD patients were incubated in 24 well culture plates at 5 · 10^5^ cells/mL for 24 hours at 37°C in a humidified 5% CO_2_/95% air atmosphere in presence of increasing concentrations (ranging from 10^−7^ M to 10^−5^ M) of original somatostatin analogs, with or without activation by LPS (5 *μ*g/mL). Compounds were dissolved in PBS. Plates were centrifuged for 15 minutes at 200 g (20°C) and supernatants stored at −20°C, prior to cytokine determination as previously described [4].

### 2.10. Statistics

Real-time RT-PCR data were quantified using the SDS 2.2.1 software package (Applied Biosystems). Results were quantified in a relative comparative study, using an automatic baseline and threshold to record the cycle thresholds (C_ts_) setting and the 18S rRNA gene expression as a reference for normalization (ΔC_ts_). Student's *t*-test was used with a significance threshold of *P* < .05.

Data from ELISA have been expressed as a percentage of the basal cytokine secretion or as means (± SE); “*n*” refers to the number of experiments. Results were compared using the nonparametric Mann–Whitney U test. The level of statistical significance was fixed at *P* < .05.

## 3. Results

By monitoring non-orphan RCPGs (i.e., 279 among the 382 RCPGs tested) in PBMCs of 10 healthy donors and 10 CD patients, we found that the data obtained were highly dependant on RNA integrity. The integrity of RNA molecules is of paramount importance for experiments that try to reflect the snapshot of gene expression at the moment of RNA extraction. The RNA integrity number (RIN) is an important tool regrettably often disregarded, in conducting valid gene expression measurement experiments as real-time PCR or DNA microarray [6]. After RIN determination of our 20 samples, only 4 CD patients and 4 paired healthy donors could be considered reliable due to sample degradation yet with handling and shipping in dry ice from the hospital to the laboratory. Thus even with only 4 CD patients and 4 controls but of reliable origins, we found that SSTR1 and 5 receptors were found differentially expressed among the 279 identified RCPGs. On the other hand, when we explored expression profiles of biopsies obtained by endoscopies from five CD patients, another pattern came out when we compared biopsies from inflamed to non-inflamed areas. This time only one receptor (CCR7) showed a significant overexpression in inflamed areas.

### 3.1. Expression of SSTR5 mRNA and CCR7 in PBMCs of CD Patients

A significant increase in SSTR5 mRNA levels (Table 1(a)) was observed in CD patients PBMCs: SSTR5 is overexpressed more than 417 ± 71 times (*P* < .05). Activation by LPS did not affect SSTR5 expression in CD patients (Table 1(b)). No difference in CCR7 mRNA expression was found in CD PBMCs when compared to those from healthy donors. 

### 3.2. Correlation of mRNA Expression with Correspondent Protein Synthesis in PBMCs

**Figure 1:**
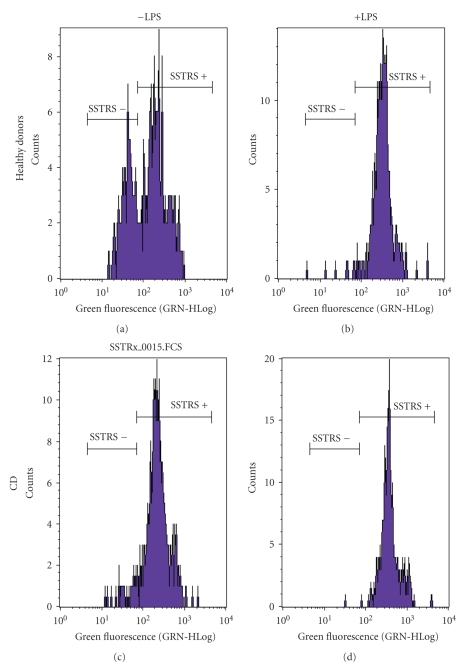
SSTR5 protein expression in PBMCs of CD patients and healthy donors activated or not by LPS. To mimic inflammation, PBMCs were incubated in presence or absence of LPS (1 *μ*g/mL) for 2 hours 30 minutes at 37°C. The cells were fixed and permeabilized and SSTR5 revealed by an Alexa 488 coupled antibody. The histogram (b) shows that after LPS activation of healthy PBMCs, all cells were presenting SSTR5 proteins, in the same way that PBMCs from CD patients activated (d) or not (c) by LPS.

Flow cytometry experiments were carried out on PBMCs to verify if protein expression corresponds to such a dramatic mRNA overexpression. The histogram in Figure 1 shows healthy PBMCs (B) with 70% exhibiting positive staining for SSTR5. After LPS activation 100% of healthy controls' PBMCs were presenting SSTR5 proteins. By contrast, in CD patients, all PBMCs showed positive staining for SSTR5 independently of activation (D) or not (C) by LPS. Nevertheless, the expected protein 400-fold SSTR5 overexpression was never seen when comparing healthy PBMCs (A) to CD patient's PBMCs (C). From these results, it appears that PBMCs from CD patients present the SSTR5 expression level of LPS-activated PBMCs from healthy donors, but no correlation could be found between the rate of mRNA and protein expression. 

Neither confocal microscopy, nor flow or capillary cytometry showed CCR7 overexpression by healthy controls or CD patients PBMCs. 

### 3.3. Deletion of 35 bp in DNA Sequence of SSTR5 in PBMCs of CD Patients

The extraction of mRNA was realized for PBMCs of 4 CD patients and 4 control donors. cDNA was obtained from RT-PCR and PCR. We found that the sequence of SSTR5 DNA in CD patients was different from control donors (Table 2). A smaller sequence in CD patients is always observed with an average difference of 35 bp. Thus the hypothesis of a deletion can be proposed. This could give a reason for CD patients to produce more SSTR5 proteins than control donors in order to supply a constant binding of somatostatin to the cells in case the protein is less efficient in ligand recognition.

### 3.4. Inhibition of the Proinflammatory Cytokine TNF-*α* by Somatostatin Analogs in PBMCs from CD Patients

**Figure 2:**
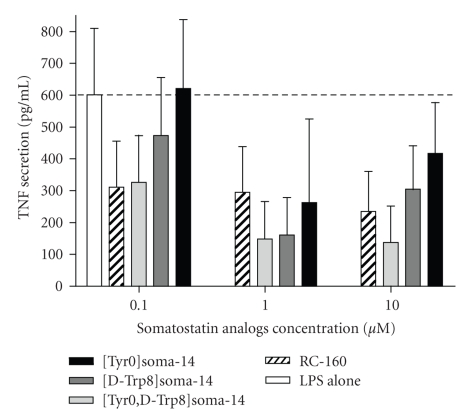
Inflammatory response (TNF-*α* secretion) of CD patient's PBMCs in presence of somatostatin analogs. CD patients PBMCs (5 · 10^5^ cells/mL) were activated by LPS for 24 hours in presence of increasing doses of somatostatin analogs (10^−7^ M to 10^−5^ M). Quantitative evaluation of the secreted cytokine TNF-*α* was done by sandwich ELISA as described in Materials and Methods.

Having found that such a high SSTR5 mRNA overexpression does not correlate to the SSTR5 protein levels in addition that CD patients present a 35 bp deletion in SSTR5 DNA, we intend to assess if the expressed SSTR5 remains active in PBMCs of CD patients. To do so, we tested the potency of different original somatostatin analogs to inhibit the production of the proinflammatory cytokine TNF-*α* after LPS activation of PBMCs obtained in CD patients (Figure 2). All four tested analogues were found active, even if in different rates, emphasizing the efficiency of the SSTR present at the surface of CD patients PBMCs.

### 3.5. Expression of CCR7 mRNA in Biopsies from CD Patients

In biopsies of intestinal tissues of CD patients, CCR7 was overexpressed by 10 ± 3 times compared to healthy donors colonic mucosa (Table 1(c)). Somatostatin receptor 5 mRNA expression was not increased in control or CD patient's intestinal mucosa.

### 3.6. Localization of CCR7 and SSTR5 in Tissues of CD Patient

**Figure 3:**
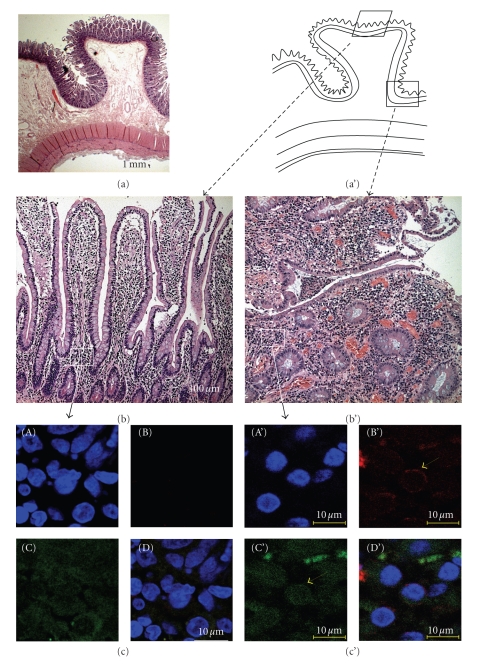
CCR7 presence in the *lamina propria* of inflamed tissues of CD patients (c') compared to the healthy donor (c) by confocal microscopy. (a) Hematoxylin/eosin coloration of healthy intestine showing the localization of the different magnifications. (b and b') Magnification of the Lieberkuhn's glands to show localization of the chorion: (b) non-inflamed tissue, (b') inflamed part. (c and c') CCR7 is detected in the inflamed CD tissues by confocal microscopy revealed by an Alexa 647 labeled secondary antibody: (c) non-inflamed *lamina propria*, (c') inflamed *lamina propria*. CCR7 staining (B and B'), nucleus staining with DAPI (A and A'), green autofluorescence (C and C') and corresponding merged images (D and D').

Immunofluorescence detection of CCR7 was not possible by classical epifluorescence techniques due to the high autofluorescence of the mucus and secretions of the intestinal tissues. It is only by confocal microscopy with an Alexa 647 labeled secondary antibody that we were able to detect CCR7 expression in the inflamed CD tissues versus non-inflamed part. Moreover, the images obtained show that the detected receptors were located at cell membrane, especially on the surface of mononuclear cells present in the intestinal lamina propria (Figure 3).

As expected, no SSTR5 labeling could be found in healthy controls or CD patients' colon biopsies, in agreement with the results obtained earlier in real time RT-PCR experiments. 

## 4. Discussion

The present work highlights through two precise examples, the importance of RNA quality assessment in data interpretation in particular that of the RNA Integrity Number (RIN) to assign integrity values to RNA. Establishment of RIN, a step often mishandled, obviously could not be replaced by examining a high number of samples. In addition, our results illustrate the fact that flow cytometry and confocal microscopy, in combination with (a) cellular challenge(s) (i.e. response to LPS activation) constitute important tools which could bring new highlights to results obtained by molecular biology experiments.

We found a significant increase in SSTR5 mRNA levels (more than 400 times) in inactive CD patients PBMCs. By contrast, flow cytometry experiments showed that SSTR5 protein expression did not reveal a protein expression levels corresponding to such a dramatic mRNA overexpression. The profile of SSTR5 protein expression in inactive CD patients' PBMCs that we observed in basal conditions (i.e., without any in vitro exogenous activation of PBMCs) was comparable to the SSTR5 expression of LPS-activated PBMCs from healthy donors. Furthermore, to answer the question of the functional activity of SSTRs from inactive CD patients, we assessed the ability of different somatostatin analogs to inhibit PBMCs TNF-*α* in vitro production. All four tested original analogues were found to be active as they significantly inhibit TNF-*α* secretion, demonstrating the efficiency of the SSTR present at the surface of CD patients PBMCs to adequately fulfill this biological property but not explaining why SSTR5 mRNA is overexpressed.

One hypothesis to explain such lack of correlation between the 400-fold increase in SSTR5 mRNA levels and the smaller increase in SSTR5 protein expression in CD patients PBMCs concerns the internalization and up-regulation of SSTR5 [8]. The cytoplasmic tail of SSTR5 is crucial for interacting with adenylyl cyclase and so in mediating desensitization and internalization [9]. Using BLAST (http://blast.ncbi.nlm.nih.gov/), we identified specific primers to amplify a locus inside the exon number 2 of SSTR5 after the promoter region. The deletion of 35 bp after amplification by PCR suggests an implication of the SSTR5 coding sequence (CDS) in CD patients. The presence of a deletion of the C-terminal tail of the GPCR may affect recycling of this GPCR by internalization and up-regulation. Thus the binding between SSTR5 and somatostatin will not be able to activate the adenylyl cyclase; as a result this may lead to favor increase in SSTR5 mRNA expression as a result of the absence of a negative retroactive regulatory loop. However, as we have shown that synthetic somatostatin analogs are still able to down regulate the TNF-*α* production of PBMCs of CD patients after LPS activation, this hypothesis remains questionable and needs additional work, both to verify if the lacking 35 bp are located in the C-terminal region, and to identify the SSTR receptor(s) involved in the inhibition of PBMCs TNF-*α* production by the used somatostatin analogues, as other SSTR receptors than SSTR5 may be involved or may compensate an hypothetical dysfunction of SSTR5.

Although it was not the aim of the present study, such an increase of SSTR5 mRNA and protein expression in PBMCs from clinically inactive CD patients, compared to healthy controls, remains intriguing. Actually, somatostatin has been shown in several studies to participate in inflammatory and immune response [10]. This role has been largely supported by the presence of somatostatin and SSTRs in cells of inflamed organs [11], structures of the immune system such as human lymphoid tissues, and finally peripheral blood cells [10]. The biological activity of somatostatin is generally of inhibitory nature. Five somatostatin receptor subtypes, termed SSTR (1–5), have been cloned so far from human tissues. SSTR (1–5) are encoded by five non-allelic genes on chromosomes 14, 17, 22, 20, and 16, respectively [12–15]. Somatostatin and somatotropin release-inhibiting factors (SRIF) are cyclopeptides. Somatostatin is produced by normal endocrine, gastrointestinal, immune, and neuronal cells, as well as by certain tumors [16–20]. The highly potent somatostatin peptides SRIF-14 and SRIF-28 are generated as C-terminal products from prosomatostatin. By binding to their receptors on target cells, SRIFs act as potent inhibitors of various secretory processes and cell proliferation [16–20]. Previous studies have demonstrated the presence of SSTRs in inflammation [21–25]. It is widely accepted that the neuropeptide somatostatin is an important regulator of the immune system in a number of tissues. There is abundance of data showing that inflammatory cytokines regulate somatostatin in immune and neural cells [26–29], and it would be interesting to explore more accurately the mechanism(s) involved and the potential role of SSTRs, for example, SSTR5 as anti-inflammatory therapeutic targets. In addition, high SSTR5 expression in PBMCs from CD showing no clinical or biological signs of activity could suggest that this SSTR5 overexpression may potentially be considered as a biomarker of CD. However, this has to be confirmed in a larger patients population, homogeneous both for disease activity and patients treatments; this work is currently ongoing. 

On the other hand, and as a second example, we studied CCR7 mRNA and protein expression in mucosal biopsies of intestinal tissues of CD patients. We found CCR7 mRNA expression to be 10-times increased compared to healthy controls colonic mucosa. This time corresponding CCR7 protein expression was detected in the inflamed CD tissues versus non-inflamed.

Chemokines also represent important actors in the control of inflammatory response. They affect cells by binding and activating surface receptors that are seven transmembrane domains GPCR. Chemokines are up regulated in inflammatory conditions and are produced by activated leukocytes and tissue cells as well as numerous tumors. They control the recruitment of effector leukocytes and thus, determine the composition of inflammatory infiltrates [30]. In this study we observed increased CCR7 mRNA expression in inflamed CD colonic mucosa, as well as a CCR7 staining in *lamina propria *mononuclear cells. These results are in accordance with data reported by Middel *et al*. [31] suggesting that in CD, lymphoid chemokines CCL20 (and its receptor CCR6) as well as CCL19 and CCL21 (and their receptor CCR7) may lead to an increased number of mature dendritic cells (DC) in the affected bowel wall, leading to local activation and proliferation of T cells, the formation of DC/T cell clusters expressing CCR7, and finally participating in the maintenance of the intestinal inflammatory and immune response. Such a “sequestration” of CCR7-positive immune cells could explain why we did not find increased CCR7 expression in PBMCs from CD patients. Whether the modulation of the lymphoid chemokines or of their receptors might represent a potent and safe therapeutic strategy remains to be clarified. If CCR7 is overexpressed in inflamed bowel of CD patients, SSTR5, an important factor in down regulating the immune response, was equally expected in the same tissue. Absence of SSTR5 in the inflamed tissue of CD patients can explain why the somatostatinergic system is not playing its immunosuppressive role in the bowel. Our data suggest that there is no need to develop new analogs of somatostatin if there is such an important lack of target expression in the inflamed bowel of CD patients.

For CD patients, we observed a difference for SSTR5 mRNA expression in circulating blood compared to biopsies. Such a difference for two distinctive localizations is difficult to explain. The high presence of SSTR5 mRNA in PBMC of CD patients could be a consequence of a diminished immune response, in turn leading to uncontrolled accumulation of the inducer stimuli thus activating the adaptive immune system [32]. We followed in parallel the expression of a panel of genes implicated in the inflammatory process. Only a slight over-expression of biomarkers like CHRM1, CCR10, FY, BDKRB1, and HTR1B (data not shown) was observed. For the over-expression of CCR7 in biopsies of CD patients, this phenomenon can be due, directly or indirectly, to a genetic defect inducing an exaggerated innate response to the intestinal microbial flora, leading to an excessive inflammatory response [33].

In conclusion, the present study highlights the need to be cautious in interpreting data only providing information on RNA expression or protein expression, in order to avoid false interpretation on receptor expression and false physiological or pathological relevance. These results emphasize also the importance of RNA quality in data analyses, in particular when using high throughput techniques like DNA chips or micro-fluidic devices. Finally, and despite it has not been the purpose of the study, we present preliminary results suggesting a role for SSTR5 and CCR7 in CD pathogenesis, which might if confirmed in further large scale studies, conduct to identify (a) new disease marker(s) and/or therapeutic target(s).

## Figures and Tables

**Table:** (a)

Assay ID	Gene Symbol	Gene Name	T-TEST	Average *⊗* *⊗*Ct
Hs00265647_s1	SSTR5	somatostatin receptor 5	0.038	9.6
Hs00171054_m1	CCR7	chemokine (C-C motif) receptor 7	0.16	-6.56

**Table:** (b)

Assai ID	Gene Symbol	Gene Name	T-TEST	Average *⊗* *⊗*Ct
Hs00265647_s1	SSTR5	somatostatin receptor 5	0.39	1.80

**Table:** (c)

Assay ID	Gene Symbol	Gene Name	T-TEST	Average *⊗* *⊗*C_ts_
Hs00171054_m1	CCR7	chemokine (C-C motif) receptor 7	0.03	3.36
Hs00265647_s1	SSTR5	somatostatin receptor 5	0.33	1.31

**Table 2:** Results of the DNA sequencing analysis of PBMCs for SSTR5 in 4 control donors and 4 CD patients. mRNA extraction was realized from PBMC of 4 CD patients and 4 control donors. cDNA was obtained after RT-PCR and PCR. Total cDNA was checked for integrity and concentration using the DNA 7500 LabChip kit on the Agilent 2100 bioanalyzer and for purity by reading the absorbance at 260 and 280 nm with ratios A260/A280 always between 1.8 and 2.0.

Donors	Size (bp)	Average size (bp) ± sd
Control 1	1284	1298 ± 10
Control 2	1310	
Control 3	1294	
Control 4	1302	

CD patient 1	1259	1263 ± 4
CD patient 2	1259	
CD patient 3	1266	
CD patient 4	1266	
